# Fatal Presentation of Leigh Syndrome in a Neonate: Comprehensive Neuroimaging Findings With MT-ND5 Mutation

**DOI:** 10.7759/cureus.96098

**Published:** 2025-11-04

**Authors:** Shrinivas Radder, Nivedita Radder

**Affiliations:** 1 Diagnostic Radiology, University of Arkansas for Medical Sciences, Little Rock, USA; 2 Pediatric Radiology, Arkansas Children's Hospital, Little Rock, USA

**Keywords:** leigh syndrome, mitochondrial disorder, mr spectroscopy, mt-nd5 mutation, neonatal encephalopathy, neuroimaging

## Abstract

Leigh syndrome represents a severe mitochondrial disorder characterized by progressive neurodegeneration, typically manifesting in infancy with devastating outcomes. We present a 30-day-old male infant who presented with acute neurological deterioration, including seizures, dystonia, and respiratory failure. Laboratory evaluation revealed elevated levels of lactate and pyruvate. Brain magnetic resonance imaging (MRI) demonstrated characteristic bilateral symmetric T2 hyperintensity with restricted diffusion involving the basal ganglia, thalami, brainstem structures, and multiple other regions. Single-voxel spectroscopy confirmed an elevated lactate peak in the basal ganglia. Genetic testing identified a 95% heteroplasmic pathogenic variant in MT-ND5, confirming mitochondrial DNA-associated Leigh syndrome. Despite intensive supportive care including mechanical ventilation and anticonvulsant therapy, the patient's condition progressively deteriorated, resulting in death 16 days after admission. This case highlights the fulminant presentation of neonatal Leigh syndrome and emphasizes the critical role of neuroimaging in establishing this diagnosis, particularly when combined with biochemical and genetic findings.

## Introduction

Leigh syndrome, also known as subacute necrotizing encephalomyelopathy, is a progressive neurodegenerative disorder that typically presents in infancy or early childhood [1]. First described by Denis Leigh in 1951, this condition results from mutations that affect mitochondrial energy metabolism, leading to characteristic, bilateral, symmetric brain lesions [2]. The disorder exhibits significant clinical and genetic heterogeneity, with over 75 different genetic causes identified, affecting both nuclear and mitochondrial DNA [3].

The incidence of Leigh syndrome is estimated at one in 40,000 live births, though this may be underestimated due to diagnostic challenges and early mortality [4]. While most cases present between three to 12 months of age, neonatal presentations, as in our case, represent a particularly severe phenotype with rapid progression and poor prognosis [5].

## Case presentation

A 30-day-old male infant was brought to our emergency department by his parents with a 48-hour history of decreased activity and poor feeding. The mother reported that the baby had been previously healthy with a normal birth history and no significant perinatal complications. Over the preceding two days, she noticed he was increasingly lethargic, refusing feeds, and had developed abnormal body movements.

On examination, the infant appeared critically ill and pale. He was unresponsive to verbal stimuli with intermittent extensor posturing of both arms and legs. Vital signs revealed tachycardia (heart rate 180 beats/minute), shallow respiratory effort with a rate of 18 breaths/minute, and hypoxemia with oxygen saturation of 60% on room air. The neurological examination revealed absent primitive reflexes, hypertonia with dystonic posturing, and a minimal response to painful stimulation. No dysmorphic features were noted.

Initial laboratory investigations revealed significant metabolic derangements as detailed in Table 1. The elevated lactate and pyruvate levels, along with an increased lactate-to-pyruvate ratio, strongly suggested mitochondrial dysfunction. The complete blood count and basic metabolic panel were otherwise within normal limits, except for compensated metabolic acidosis.

**Table 1:** Laboratory findings at presentation

Parameter	Patient Value	Reference Range	Units
Blood lactate	8.2	<2.0	mmol/L
Blood pyruvate	0.35	0.08-0.15	mmol/L
Lactate/pyruvate ratio	23.4	10-20	-
pH (arterial)	7.28	7.35-7.45	-
pCO2	32	35-45	mmHg
HCO3-	15	22-28	mEq/L

Neuroimaging findings

**Figure 1:**
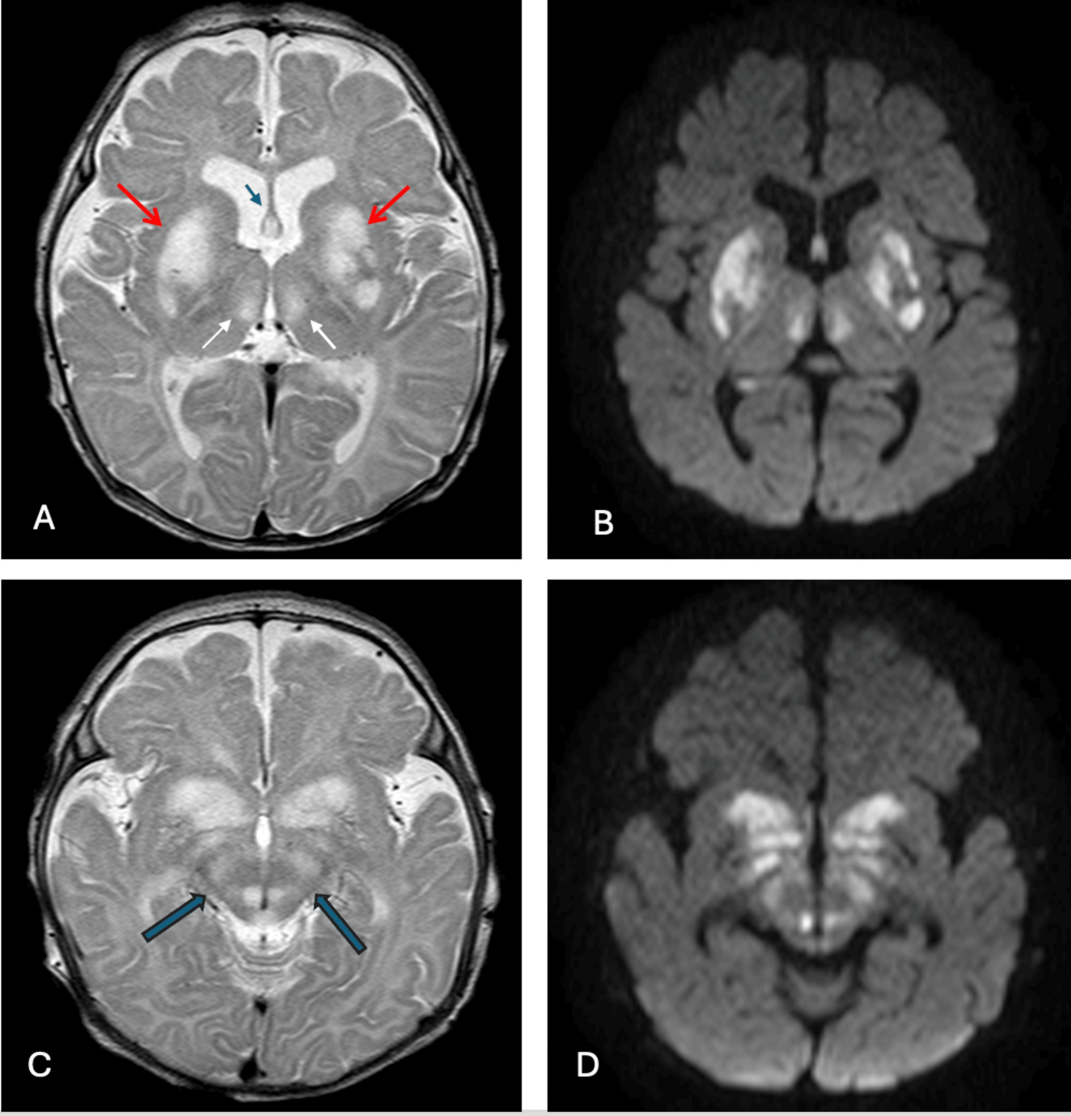
Brain magnetic resonance imaging findings. (A) Axial T2-weighted sequence demonstrates bilateral symmetric hyperintense signal abnormalities within the lentiform nuclei (red arrows) and medial thalami (white arrows). Additional areas of T2 hyperintensity are noted in the perirolandic cortex, fornix (thin blue arrow), hypothalamus, bilateral hippocampi, and splenium of the corpus callosum (regions not shown). (B) Corresponding axial diffusion-weighted imaging shows restricted diffusion in the same anatomical distributions. (C) Axial T2-weighted image at the midbrain level reveals symmetric hyperintense signal changes involving the cerebral peduncles, substantia nigra, and periaqueductal gray matter (thick blue arrows). (D) Axial diffusion-weighted imaging at the midbrain level confirms restricted diffusion within these brainstem structures, consistent with acute metabolic injury.

Brain MRI performed on hospital day two revealed extensive bilateral symmetric abnormalities. T2-weighted and fluid-attenuated inversion recovery (FLAIR) sequences demonstrated hyperintense signal involving the lentiform nuclei, medial thalami, perirolandic regions, fornix, hypothalamus, hippocampi, and splenium of the corpus callosum (Figure 1A, 1B). The midbrain showed symmetric involvement of the cerebral peduncles, substantia nigra, and periaqueductal gray matter (Figure 1C, 1D). Diffusion-weighted imaging confirmed restricted diffusion in these regions, suggesting acute metabolic injury. No hemorrhage or calcification was identified on gradient echo sequences, and post-contrast images showed no abnormal enhancement.

**Figure 2:**
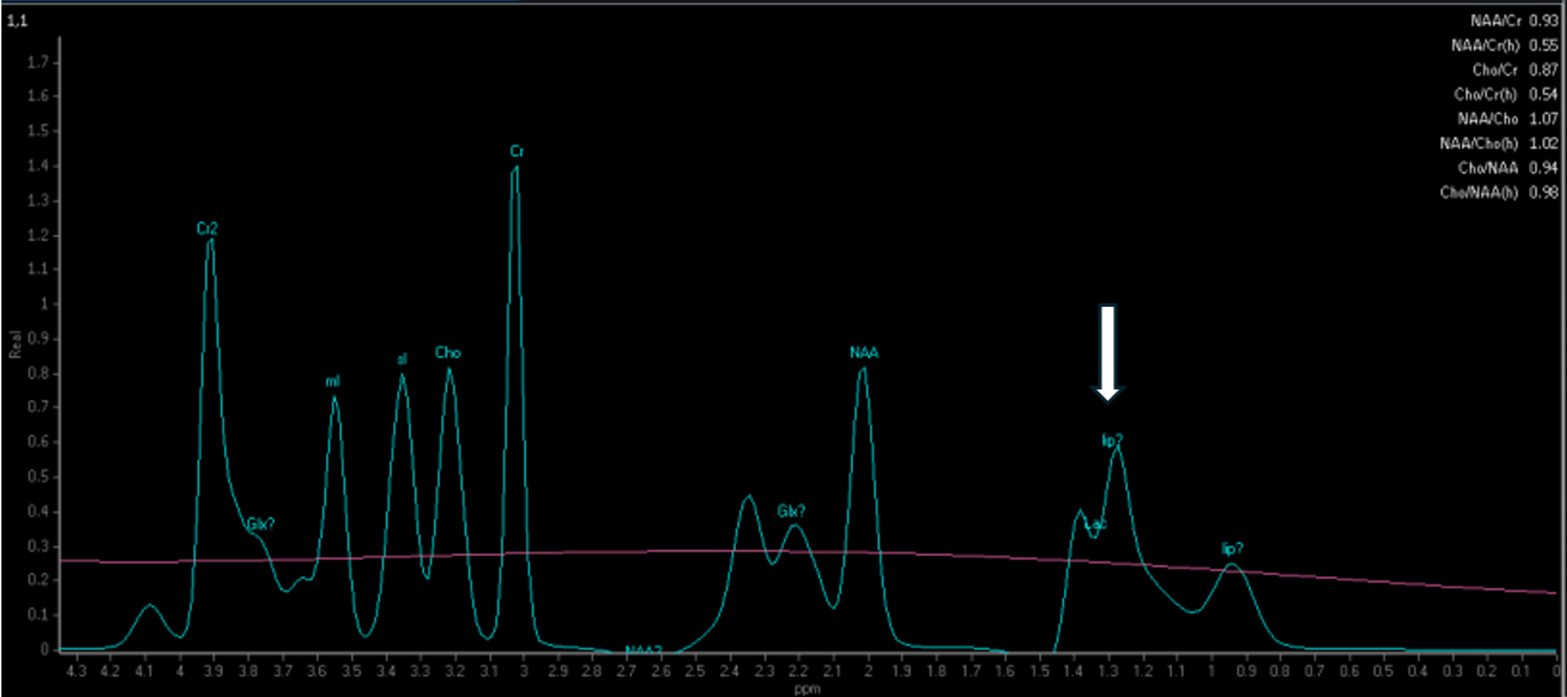
Single-voxel magnetic resonance spectroscopy of the right basal ganglia demonstrates a prominent inverted doublet peak at 1.33 ppm (arrow), indicating pathologically elevated lactate concentration within the affected brain parenchyma. The presence of lactate reflects impaired mitochondrial oxidative metabolism characteristic of Leigh syndrome. Note the reduced N-acetylaspartate peak at 2.0 ppm, suggesting neuronal loss or dysfunction in this region.

Single-voxel MR spectroscopy (Figure 2) centered on the right basal ganglia demonstrated a prominent inverted doublet peak at 1.33 ppm, confirming elevated lactate levels within the affected brain parenchyma. The N-acetylaspartate peak was diminished, suggesting neuronal loss.

Additional investigations

Genetic testing using next-generation sequencing of mitochondrial DNA identified a pathogenic variant in MT-ND5 (m.13513G>A) with 95% heteroplasmy, confirming the diagnosis of mitochondrial DNA-associated Leigh syndrome.

Transthoracic echocardiography findings were obtained from the official cardiology report in the electronic medical record. The study revealed mild stenosis of the right pulmonary artery with a peak gradient of 18 mmHg. Left ventricular systolic function was preserved with an ejection fraction of 65%. No ventricular hypertrophy, pericardial effusion, or other structural abnormalities were identified.

Clinical course

The patient was intubated for respiratory support and started on phenobarbital for seizure control. Despite aggressive supportive care including mechanical ventilation, anticonvulsant therapy, and metabolic supplementation with thiamine, riboflavin, and coenzyme Q10, his condition progressively deteriorated. He developed refractory hypotension requiring vasopressor support and persistent hyperkalemia despite medical management. Continuous EEG monitoring showed severe background suppression with multifocal epileptiform discharges. The patient died on hospital day 16 following cardiorespiratory arrest.

## Discussion

This case illustrates the devastating neonatal presentation of Leigh syndrome with characteristic neuroimaging findings and confirmed MT-ND5 mutation. The symmetric involvement of basal ganglia, thalami, and brainstem structures on MRI, combined with elevated lactate on both serum analysis and MR spectroscopy, provided crucial diagnostic information that guided genetic testing [6].

The MT-ND5 gene encodes a subunit of complex I, a component of the mitochondrial respiratory chain. Mutations in this gene account for approximately 10-20% of cases of mitochondrial DNA-associated Leigh syndrome [7]. The high heteroplasmy level (95%) in our patient correlates with the severe clinical phenotype and early presentation, as tissue-specific mutation load directly influences disease severity [8].

Neuroimaging plays a pivotal role in diagnosing Leigh syndrome, particularly in acute presentations where rapid clinical decision-making is essential. The bilateral symmetric pattern of involvement, particularly affecting the basal ganglia and brainstem, distinguishes Leigh syndrome from other metabolic and hypoxic-ischemic encephalopathies [9]. MR spectroscopy adds specificity by demonstrating elevated lactate within affected brain regions, reflecting impaired oxidative metabolism.

The neonatal presentation of Leigh syndrome, as seen in our case, carries an exceptionally poor prognosis. Most affected infants die within months of symptom onset, typically from respiratory failure due to brainstem involvement [10]. While supportive care, including cofactor supplementation, may provide temporary stabilization, no curative treatment currently exists for Leigh syndrome.

## Conclusions

This case emphasizes the importance of considering Leigh syndrome in neonates presenting with acute encephalopathy, metabolic acidosis, and elevated lactate levels. The characteristic neuroimaging pattern on MRI, particularly when combined with MR spectroscopy showing elevated brain lactate, should prompt immediate genetic testing for mitochondrial disorders. Early diagnosis, while not altering the outcome in severe cases, enables appropriate family counseling and genetic risk assessment for future pregnancies.
